# Evaluation of two commercial IBR marker vaccines against *Bubaline alphaherpesvirus 1* in water buffalo (*Bubalus bubalis*)

**DOI:** 10.3389/fvets.2025.1574794

**Published:** 2025-05-07

**Authors:** Giovanna Cappelli, Stefano Petrini, Francesco Grandoni, Carlo Grassi, Immacolata De Donato, Federica Signorelli, Francesco Napolitano, Roberta Vecchio, Anna Balestrieri, Esterina De Carlo, Giovanna De Matteis, Alessandra Martucciello

**Affiliations:** ^1^National Reference Centre on Water Buffalo Farming and Productions Hygiene and Technologies (CReNBuf), Istituto Zooprofilattico Sperimentale del Mezzogiorno, Portici, Italy; ^2^National Reference Centre for Infectious Bovine Rhinotracheitis (IBR), Istituto Zooprofilattico Sperimentale Umbria-Marche “Togo Rosati”, Perugia, Italy; ^3^Research Centre for Animal Production and Aquaculture, Consiglio per la Ricerca in Agricoltura e l’Analisi dell’Economia Agraria (CREA), Monterotondo, Italy

**Keywords:** water buffalo, IBR, marker vaccines, BoAHV-1, BuAHV-1

## Abstract

The present study aimed to evaluate two commercial infectious bovine rhinotracheitis (IBR) marker vaccines against *Bubaline alphaherpesvirus 1* (BuAHV-1) in water buffalo *(Bubalus bubalis)*. Thirteen water buffaloes seronegative to Bovine alphaherpesvirus 1 (BoAHV-1) and BuAHV-1 were selected and divided into three groups (VAX-1, VAX-2, CNT). VAX-1 received an IBR marker (gE-/tk-) live vaccine; VAX-2 received an IBR marker (gE-) inactivated vaccine; CNT represented the controls. Two injections of 2 mL each were administered 21 days apart. On 55 post-vaccination days (PVDs), all animals were challenged infected with wild-type BuAHV-1. Nasal swabs and serum samples were collected at different experimental times and were used for virological, serological and immunological investigations. After seven post-challenge days (PCDs), only the CNT evidenced nasal mucus discharge and increased rectal temperature. The glycoprotein B (gB) of BoAHV-1 positivity was detected using Real-time PCR from PCDs 2 to 7 in vaccinated groups. In the controls, gB positivity was detected from PCD 2 to 15. On PVD 34, all vaccinated animals progressively increased their neutralizing antibody (NA) titers statistically until the end of the experiments. In the controls, the NAs appeared on PCD 10. Flow cytometric analysis of lymphocyte populations revealed that BuAHV-1 activates adaptive immune responses. Throughout the entire examination period, both vaccinated and unvaccinated animals exhibited similar trends. However, significant differences were observed at specific time points in the CD4^+^, CD8^+^, and γδ T lymphocyte subsets between the vaccinated groups and control group. These findings suggested that the IBR marker vaccines tested in this study could be used to protect the water buffalo against BuAHV-1.

## 1 Introduction

In Italy, water buffalo (*Bubalus bubalis*) is zootechnically and economically relevant in connection with a typical renowned product, Mozzarella di Bufala Campana (1). This species is mainly reared in southern Italy, particularly in the Campania Region, with an animal population of 305,023 heads (2). Water buffaloes and cattle (*Bos taurus*) are closely related species as they belong to the same family, *Bovidae*. However, they belong to different genera, which highligths their dissimilarities. Thus, research on cattle cannot be applied to water buffaloes without proper verification. The susceptibility of hosts to different herpesviruses has somehow changed over the years. Both water buffaloes and cattle are now known to be susceptible to both *Bovine alphaherpesvirus 1* (BoAHV-1) and *Bubaline alphaherpesvirus 1* (BuAHV-1) (3). These viruses have gone through a process of “adaptation” to ensure a higher survival rate in the environment, even after a latent infection. The contagion occurs horizontally and vertically (4), causing both direct (clinical or subclinical disease) and indirect (infertility, decreased milk production, abortion, encephalitis) health damages in animals. The infection explains why IBR is a disease of great economic importance in many parts of the world, particularly in Europe (5–10). In European countries, our IBR can negatively affect the marketing and movement of seropositive animals from one area to another (6). To date, however, the cost of IBR is a missing piece of information in Europe. In one study, it was shown that inoculating 5-month-old water buffaloes with a wild-type BoAHV-1 isolated from a bovine outbreak resulted in water buffaloes being susceptible to BoAHV-1 infection (7). In addition, the importance of this species as a reservoir for BoAHV-1 has been evidenced. However, the presence of BoAHV-1 in water buffaloes is not associated with the severe clinical signs usually seen in cattle infections, especially where cattle and water buffaloes are kept together on the same farm. On the contrary, different clinical signs (breathing difficulty, coughing, sneezing, wheezing, nasal and conjunctival hypersecretion, hyperthermia > 41°C, inappetence, sensory depression and lethargy) were evidenced in a study with young water buffalo calves infected with BuAHV-1. In some subjects, enteric symptoms (diarrhea) were observed and one water buffalo calf showed hair ruffling (9). For these reasons and due to the lack of a specific water buffalo vaccine against IBR, the present study aimed to evaluate two commercial IBR marker vaccines against *Bubaline alphaherpesvirus 1* (BuAHV-1) in water buffalo *(Bubalus bubalis).*

## 2 Materials and methods

### 2.1 IBR marker vaccines

The IBR marker vaccines used in this study were: (a) live attenuated IBR marker vaccine (gE-/tk-; VAX-1); (b) inactivated IBR marker (gE-; VAX-2). Both IBR marker vaccines were administered intranasally (i.n.), while the booster doses were injected intramuscularly (i.m.) into the neck muscle 21 days thereafter. Each vaccine was administered at a dose of 2 mL/head. This concentration is authorized for the bovine species. As there is no indication for the buffalo species, we used the same concentrations authorized for cattle in this study (Table 1).

**Table 1:** IBR marker vaccines used in the experiment.

No. of water buffaloes	Vaccine identification	Type	Virus concentration TCID_50_ in One dose (2 mL)	No. of inoculation	Inoculation route
4	VAX-1	Live IBR-marker vaccine (gE-/tk-)	5 × 10^5.50^ TCID_50_/mL	2	Intranasal (first immunization)/Intramuscular (Booster)
4	VAX-2	Inactivated IBR marker vaccine (gE-)	5 × 10^5.50^ TCID_50_/mL	2	Intranasal (first immunization)/Intramuscular (Booster)
5	CNT	*Unvaccinated control*			

gE, glycoprotein E of Bovine alphaherpesvirus-1; tk, thymidine-kinase enzyme of Bovine alphaherpesvirus-1; TCID_50_/mL, median Tissue Culture Infectious Dose.

### 2.2 Virus

Two viruses were selected for the present study. The Schönböken strain of BoAHV-1 was used for *in vitro* testing. Prof. Martin Beer, Friedrich-Loeffler Institute, Greifswald, Germany, kindly provided the BoAHV-1. In addition, the wt-BuAHV-1 strain was used for *in vitro* and *in vivo* infections (11) (GenBank accession No. KF679678.1). Viruses were cultured in Madin-Darby Bovine Kidney (MDBK) cells at a median tissue culture infection dose of 1.5 × 10^8.00^ TCID_50_/mL, calculated by the Reed and Muench method (12). All *in vitro* experiments with BuAHV-1 and BoAHV-1 cells were performed in a biosafety level 2 (BSL2) laboratory.

### 2.3 Experimental design

A linear model with ANOVA procedure was used to estimate the number of animals to be used. Using the variance and minimum difference obtained and assuming a significance level of 0.05 relative to a value of t = 1.987 corresponding to 88 degrees of freedom of error, n was estimated using the formula *n* > = 2 * Ϭ2 * (tα; v / d0)2. Thirteen three-month-old water buffalo calves free of neutralizing antibodies (NA) to BoAHV-1 and BuAHV-1 were selected, according to the results obtained in a previous study (13). The purchased animals came from two farms in Italy, one in the Lazio Region (Central Italy) and one in the Calabria Region (Southern Italy). According to the herd’s history, a BoAHV-1 vaccine had never been used on both farms, and no clinical symptoms that could be traced back to herpesvirus infection had ever been diagnosed. The water buffaloes were transferred to the experimental farm enclosure of the Istituto Zooprofilattico Sperimentale del Mezzogiorno (IZSM) after obtaining proper authorization (No. 202/2021-PR) under Directive 2010/63/EU on the protection of animals used for scientific purposes released from the Italian Ministry of Health. Animals in each group were housed in separate paddocks, were fed grass hay *ad libitum*, pelleted feed of 5 kg/head/day and had free access to water.

### 2.4 Immunization and challenge infection

The water buffaloes were divided into three groups: VAX-1, VAX-2 and CNT as unvaccinated control group. All animals were clinically examined and rectal temperature measured daily from 1 month before the immunization to 63 post-challenge days (PCDs). In addition, all animals were sampled through nasal swabs, dry and in MEM medium. Samples were collected at 0, 14, 20 and 34 post-vaccination days (PVDs) and at 0, 2, 4, 7, 10, 15, 30, and 63 PCDs. Fifty-five days after the first vaccination, all animals were challenge infected with a wild-type BuAHV-1 strain (GenBank accession number KF679678.1). The virus was injected into each animal by intranasal route at a 5 × 10^5.50^ TCID_50_/mL dose. At 0, 2, 4, 7, 10, 15, 20, 30, and 63 PCDs, nasal swabs and serum samples were collected from all animals. Two RT-PCRs against BoAHV-1 and BuAHV-1 (see Section 2.6) were performed on nasal swab samples. In addition, ELISA tests (see Section 2.8) and virus neutralization assays (see Section 2.8) against BoAHV-1 and BuAHV-1 were performed on serum samples.

### 2.5 Clinical monitoring

The animals in each group were housed in separate pens with free access to food and water. The water buffaloes were clinically monitored throughout the experimental period by measuring rectal temperature and assessing their general clinical condition (presence of nasal and ocular discharge; detecting respiratory and enteric symptoms; palpating explorable lymph nodes). The clinical signs were monitored and humane endpoints (HEP) were evaluated using the ‘Working paper on a severity assessment framework’ by an expert European group to assess the severity suffered by animals subjected to scientific procedures.

### 2.6 Virological investigations

The commercial QIA Symphony DSP Virus/Pathogen Mini Kit (Qiagen, Hilden, Germany) was used to extract viral nucleic acid from nasal swabs according to the manufacturer’s instructions. Real-time PCR was used to amplify the highly conserved target region of the UL27 gene, which is common to all alphaherpesviruses and encodes glycoprotein B (gB) of BoAHV-1 (14). All samples were tested in duplicate for analysis. Positive (IBR Los Angeles strain) and negative (PBS) controls were included in the procedure. The protocol provided the addition of an internal amplification control (*β*-actin). The sample was considered positive if the cycle threshold (Ct) was ≤ 45. In addition, 100 μL of each nasal swab was plated into three wells of a 24-well plastic plate (CytoOne® Plate; Starlab LTD, Blakelands, United Kingdom) containing monolayers of MDBK cell cultures grown in MEM. The MDBK cells were provided by the Biobank of Veterinary Resources (BVR; Brescia, Italy) and were identified as BS CL 63. After incubation for 60 min at 37°C in a 5% CO_2_ atmosphere, 1 mL of MEM supplemented with 2% fetal calf serum (BioWhittaker Inc., Walkersville, MD, United States) was added to each well. As positive controls, MDBK cells infected with BuAHV-1 were used. MDBK cell cultures free of BuAHV-1 were used as negative controls. The plates were incubated for 7 days at 37°C in an atmosphere of 5% CO_2_ and were observed daily to determine whether a cytopathic effect (CPE) occurred. Viral titers were determined by the Reed and Muench method (12) and expressed as total tissue culture infectious doses (TCID_50_/mL).

### 2.7 Serological surveys

The detection of antibodies against glycoprotein B (gB) and glycoprotein E (gE) of BoAHV-1 was performed using different commercial ELISA tests (ID Screen® IBR gB competition, and ID Screen® IBR gE competition; both from Innovative Diagnostics, Grables, France). In addition, the discrimination ELISA test BoAHV-1/BuAHV-1 was used (Eradikit™ BoAHV-1-BuAHV-1 Discrimination Kit; In3Diagnostic). All data were analyzed using Microplate Manager version 6 software (Bio-Rad Laboratories S.r.l., Segrate, Italy). The results were computed according to the manufacturer’s instructions. Virus neutralization (VN) test was carried out by applying the protocol described in the WOAH Manual of Diagnostic Tests and Vaccines (14–23). Briefly, 50 μL of 100 TCID_50_/ml of the virus was mixed with 50 μL of undiluted serum samples using two-fold dilutions for each serum sample. The tests with BuAHV-1 and BoAHV-1 (see Section 2.2.) were carried out in parallel and in separate working sessions. Each mixture was then dispensed into three wells of 96-well microtitre plates (Nunc™, 96-Well Microplates Polypropylene, Thermo Scientific, Milan, Italy). After incubation at 37°C for 24 h, 30,000 MDBK cell cultures suspended in 100 μL MEM were added to each well and incubated at 37°C for 4 days. The plates were then incubated and examined for cytopathology using a tissue culture microscope (Zeiss Axiovert Vert. A1, Zeiss International, Milan, Italy). Neutralization titers were expressed as the maximum dilution that inhibited the cytopathic effect.

### 2.8 Hematological and flow cytometry analysis

**Figure 1:**
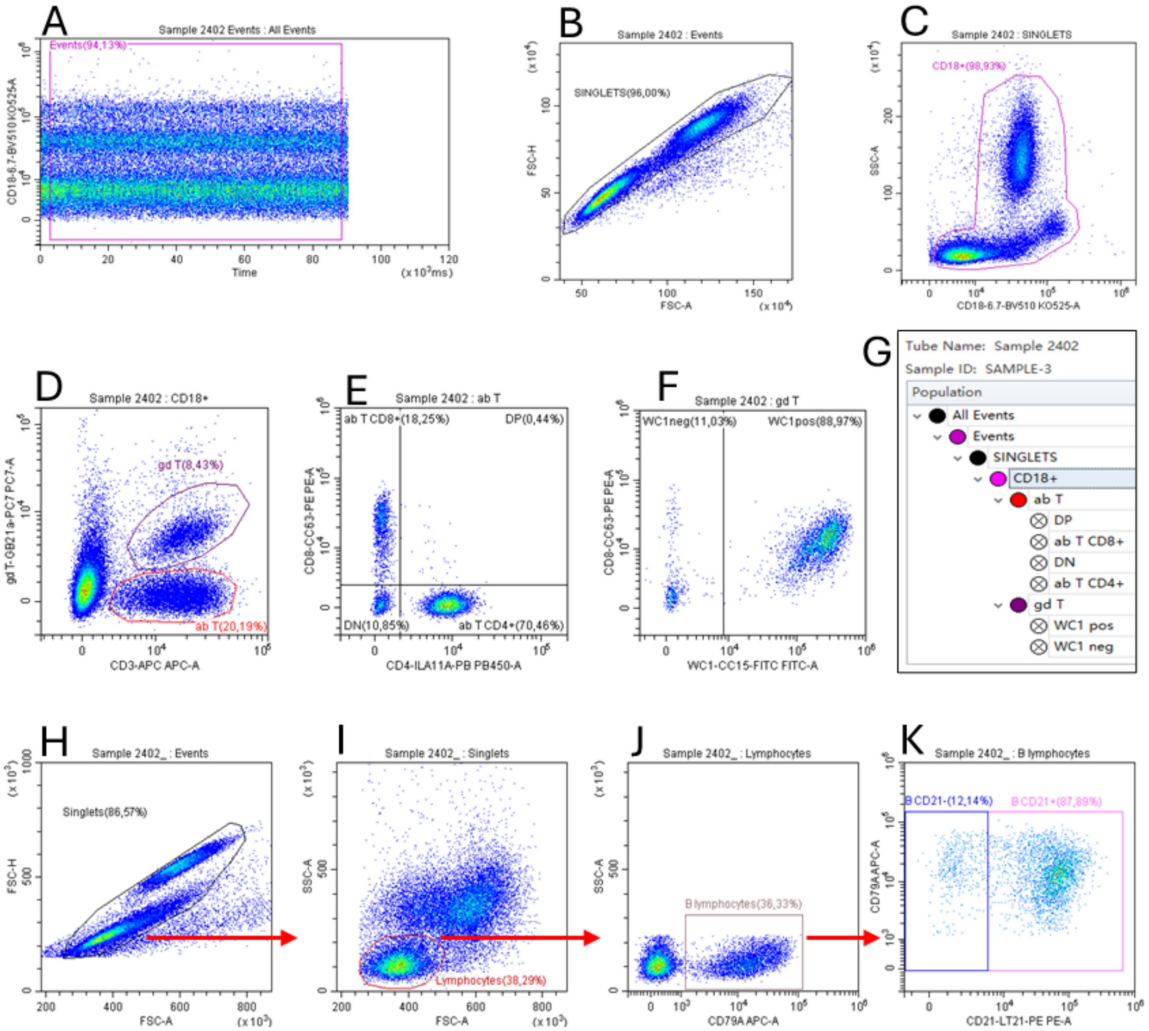
Flow cytometric gating strategy used to identify T **(A–G)** and B lymphocyte **(H–K)** subsets. The gate “Events” on dot plot Time vs. FL **(A)** was used to exclude event burst. The gate “Singlets” (FSC-A vs. FSC-H) was used to exclude doublets **(B,H)**, while the gate “CD18^+^” (CD18 vs. SSC) was used to identify total leukocytes **(C)**. Applying this gate to the CD3 vs. gd T dot plot **(D)**, we identified the αβ T (as CD3^+^/*δ* chain^−^ cells, see section 2.7) and γδ T lymphocyte subsets (as CD3^+^/δ chain^+^). The gate “ab T” was applied to the CD4 vs. CD8 dot plot to identify these subsets **(E)**. The gate “gd T” was applied to the WC1 vs. CD8 dot plot to identify the WC1^+^ γδ T lymphocyte subset. The gate hierarchies are shown in the G box. The Time vs. FL dot plot, used to exclude event burst, is not shown for the two-color panel used for B lymphocytes **(H–K)**. To identify these cells, singlet events **(H)** were used in the FSC vs. SSC dot plot **(I)** to create the gate “Lymphocytes” that applied to the dot plot CD79A vs. SSC **(J)** allowed us to identify total B lymphocytes as CD79a^+^ cells. Finally, the CD21^+^ B lymphocytes were identified in the dot plot CD21 vs. CD79A **(K)**.

Hematological and flow cytometry analyses were performed on whole blood samples collected from the jugular vein, respectively, into K_3_EDTA and Li-Heparin tubes (Vacuette®, Greiner Bio-One, Rome, Italy). Hematological analyses were determined by the Cell-Dyn 3,700 SL instrument (Abbott, Abbott Park, IL, United States), according to the standard operating procedure. Since the leukocyte viability, assessed by LIVE/DEAD Fixable Near-IR stain kit® (Thermo Fisher Scientific, Waltham, MA, United States) was always higher than 98% (data not shown), B and T lymphocyte subsets were evaluated by flow cytometry using two different multicolor panels without live/dead discrimination. To evaluate the T lymphocyte subsets, a six-color panel was performed: anti-CD3 AF647 (clone MM1a); anti-CD4 Pacif-ic Blue (clone IL-A11a); anti-CD8 PE (clone CC63), anti-CD18 Brillant Violet 510 (clone 6.7); anti-*δ* chain PE-Cy7 (clone GB21a), anti-WC1 FITC (clone CC15). A two-color flow cytofluorimetric panel was used to identify B lymphocytes: anti-CD21 PE (clone LT21) and anti-CD79a APC (clone HM47). The sample staining conditions were performed as described in our previous study (13). After staining, samples were immediately acquired on a CytoFLEX flow cytometer, and the post-acquisition data were analyzed by CytExpert 2.4 software (Beckman Coulter, Brea, CA, United States). The gating strategy used to identify the sub-sets of interest is described in Figure 1. The lymphocyte subset absolute counts were estimated by combining flow cytometric relative percentages and lymphocyte absolute count obtained by hematological analysis.

### 2.9 Statistical examination

A logarithmic scale of base 10 was used to express mean neutralizing antibody titers. Statistically significant differences were assessed using the Shapiro–Wilk and the non-parametric Wilcoxon-Mann–Whitney tests. The comparison was made between:

1. Neutralizing antibodies (NAs) against BuAHV-1 between vaccination groups (VAX-1 and VAX-2) and control groups (CNT);
2. NAs against BoAHV-1 between vaccination groups (VAX-1 and VAX-2) and control groups (CNT).

The level of significance was set at *p* < 0.05.

Hematologic and flow cytometric data were analyzed using the PROC MIXED procedure of SAS 9.4 (SAS Institute Inc., Cary, CA, United States) as follows:

$$Y_{\mathrm{ijkl}}=\mu +G_{i}+D_{j}+{GD}_{ij}+b_{jk}+\varepsilon_{\mathrm{ijkl}}$$

where Y_ijkl_ is the dependent variable; μ is the overall mean; G_i_ is the fixed effect of the ith group (CNT, VAX-1, and VAX-2); Dj is the fixed effect of the jth PCDs 0, 2, 4, 7, 10, 15, 30, and 63; GD_ij_ is the fixed effect of the interaction between the ith group and jth day; b_jk_ is the random effect of the subject within the time j (k = 1, …,13); and ε_ijkl_ is the random error. Differences were considered significant at *p* < 0.05.

## 3 Results

### 3.1 Clinical response

During the entire vaccination period, no clinical signs or adverse reactions were observed in immunized animals. After the challenge infection, no lesions were detected in immunized animals. Only in the control group at PCDs 7–14 four out of five water buffaloes showed nasal mucus discharge, while in one animal, a nasal mucous membrane lesion associated with mucopurulent exudate and blood was detected; in addition, in the control group, an increase in rectal temperatures with a maximum peak of 39.1°C was observed from 0 to 63 days.

### 3.2 Virological investigations

During the vaccination period all animals were virologically negative. On the contrary, after the challenge infection, gB positivity was detected by Real-time PCR in VAX-1 and VAX-2 groups from PCDs 2 to 7. The same positivity was observed in CNT, from PCDs 2 to 15 (Table 2).

**Table 2:** Results obtained by gB Real-time PCR after challenge infection with wild-type BuAHV-1 strain.

Group	Post-challenge day (PCD)							
	0^*^	2	4	7	10	15	30	63
VAX-1	-	4^	3	1	-	-	-	-
VAX-2	-	4	4	1	-	-	-	-
CNT	-	5	5	4	2	2	0	0

^*^PCD 0 corresponds to PVD 55; -, negative results; ^, number of water buffaloes resulting positive with a cycle threshold (Ct) equal to or less than 45.

Regarding the viremia, the virus was isolated from all groups PCDs 2 to 7 (Table 3).

**Table 3:** Results obtained by virus isolation after challenge infection with wild-type BuAHV-1 strain.

Group	Post-challenge day (PCD)							
	0^*^	2	4	7	10	15	30	63
VAX-1	-	4.50 (4)^a,b^	3.24 (3)	1.00 (1)	-	-	-	-
VAX-2	-	4.50 (4)	3.50 (4)	1.00 (1)	-	-	-	-
CNT	-	5.00 (5)	4.50 (5)	2.74 (4)	-	-	-	-

^*^PCD 0 corresponds to PVD 55; -; negative results; ^a^Reciprocal value of the negative log of the TCID_50_/mL (group mean value); ^b^The number of water buffaloes from which the BuAHV-1 was isolated is shown in brackets.

### 3.3 Serological surveys

VAX-1 group showed antibodies to gB-ELISA at PVD 14, whereas the VAX-2 group evidenced the same antibodies at PVD 34. In contrast, during the immunization period, the control group resulted negative in all ELISA tests (Table 4). An increase in NA BoAHV-1 titers at PVD 14 in the VAX-1 group was observed. In the VAX-2 group, an increase of NA BoAHV-1 titers was detected at PVD 34. A comparison of the results for NA BuAHV-1 evidenced antibodies PVD 14 in only the VAX-1 group. At PVD 34, an increase of NA BuAHV-1 was observed in the VAX-1 and VAX-2 groups compared to the control group. In contrast, the ELISA discrimination kit Bo/Bu and gE-ELISA did not detect antibodies during the entire vaccination period. No antibody changes were observed during the entire vaccination period in the CNT group (Table 4).

**Table 4:** Antibody response of water buffaloes immunized against BoAHV-1 with IBR marker vaccine.

Group	Test	Post-vaccination days (PVD)		
		0	14	34
VAX-1	gE-ELISA^a^	−	−	−
	gB-ELISA^b^	−	+	+
	ELISA Bo/Bu^c^	−	−	−
	NA BoAHV-1	<1.00	0.38	1.88
	NA BuAHV-1	<1.00	0.15	1.58
	*p*-value NA BoAHV-1	1	0.3333	0.0159
	*p*-value NA BuAHV-1	1	0.8889	0.0159
VAX-2	gE-ELISA^a^	−	−	−
	gB-ELISA^b^	−	−	+
	ELISA Bo/Bu^c^	−	−	−
	NA BoAHV-1	<1.00	<1.00	0.53
	NA BuAHV-1	<1.00	<1.00	0.45
	*p*-value NA BoAHV-1	1	1	0.0952
	*p*-value NA BuAHV-1	1	1	0.1429
CNT	gE-ELISA^a^	−	−	−
	gB-ELISA^b^	−	−	−
	ELISA Bo/Bu^c^	−	−	−
	NA BoAHV-1	<1.00	<1.00	<1.00
	NA BuAHV-1	<1.00	<1.00	<1.00

^aID Screen® IBR gB competition Innovative Diagnostics, Grables, France; bID Screen® IBR gE competition Innovative Diagnostics, Grables, France; cEradikit™ BoAHV-1-BuAHV-1 Discrimination Kit, In3Diagnostic; p-value indicating the significant differences in NA titer between vaccinated buffaloes and unvaccinated controls.^

After challenge infection, the vaccinated groups maintained the positivity to gB-ELISA until the end of the experiments. In addition, VAX-1 and VAX-2 groups produced specific antibodies to gE-ELISA at PCD 63. Moreover, a progressive increase of NA BoAHV-1 and NA BuAHV-1 was observed until the end of the experiment. In contrast, the ELISA discrimination kit Bo/Bu detected antibodies (against BoAHV-1 and BuAHV-1) in immunization groups PCD 30, whereas PCD 15 was detected in the CNT. On the contrary, the CNT group developed antibodies to gB and gE at PCDs 10 and 15, respectively. Also, in the CNT group, NA BoAHV-1 and NA BuAHV-1 were produced at PCD 10 (Table 5).

**Table 5:** Antibody response of water buffaloes immunized against BoAHV-1 with IBR marker vaccines.

Group	Test	Post-challenge day (PCD)							
		0^*^	2	4	7	10	15	30	63
VAX-1	gE-ELISA^a^	−	−	−	−	−	−	−	+
	gB-ELISA^b^	+	+	+	+	+	+	+	+
	ELISA Bo/Bu^c^	−	−	−	−	−	−	+	+
	NA BoAHV-1	1.81	1.88	1.88	2.11	2.11	2.41	2.41	3.01
	NA BuAHV-1	1.81	1.88	1.88	2.41	2.41	2.71	2.71	3.39
	*p*-value NA BoAHV-1	**0.0159**	**0.0159**	**0.0159**	**0.0159**	**0.0159**	**0.0159**	**0.0159**	**0.0159**
	*p*-value NA BoAHV-1	**0.0159**	**0.0159**	**0.0159**	**0.0159**	**0.0159**	**0.0159**	**0.0159**	**0.0317**
VAX-2	gE-ELISA^a^	−	−	−	−	−	−	−	+
	gB-ELISA^b^	+	+	+	+	+	+	+	+
	ELISA Bo/Bu^c^	−	−	−	−	−	−	+	+
	NA BoAHV-1	0.98	1.05	0.98	1.81	1.88	2.11	2.26	2.63
	NA BuAHV-1	0.45	0.60	0.68	2.11	2.11	2.41	2.63	2.94
	*p*-value NA BoAHV-1	**0.0159**	**0.0159**	**0.0159**	**0.0159**	**0.0159**	**0.0159**	**0.0159**	0.1429
	*p*-value NA BoAHV-1	0.8889	0.9520	0.3333	**0.0159**	**0.0159**	**0.0159**	**0.0317**	0.1905
CNT	gE-ELISA^a^	−	−	−	−	−	+	+	+
	gB-ELISA^b^	−	−	−	−	+	+	+	+
	ELISA Bo/Bu^c^	−	−	−	−	−	+	+	+
	NA BoAHV-1	0.0	0.0	0.0	0.0	0.060	0.90	1.44	2.11
	NA BuAHV-1	0.0	0.0	0.0	0.0	0.24	1.32	2.05	2.41

^*^PCD 0 corresponds to PVD 55; ^a^ID Screen® IBR gB competition Innovative Diagnostics, Grables, France; ^b^ID Screen® IBR gE competition Innovative Diagnostics, Grables, France; ^c^EradikitTM BoAHV-1-BuAHV-1 Discrimination Kit, In3Diagnostic; *p*-value indicating the significant differences in NA titer between vaccinated buffaloes and unvaccinated controls. Statistically significant values are in bold.

### 3.4 Flow cytometric profiling of bubaline lymphocyte subsets during BuAHV-1 infection

**Figure 2:**
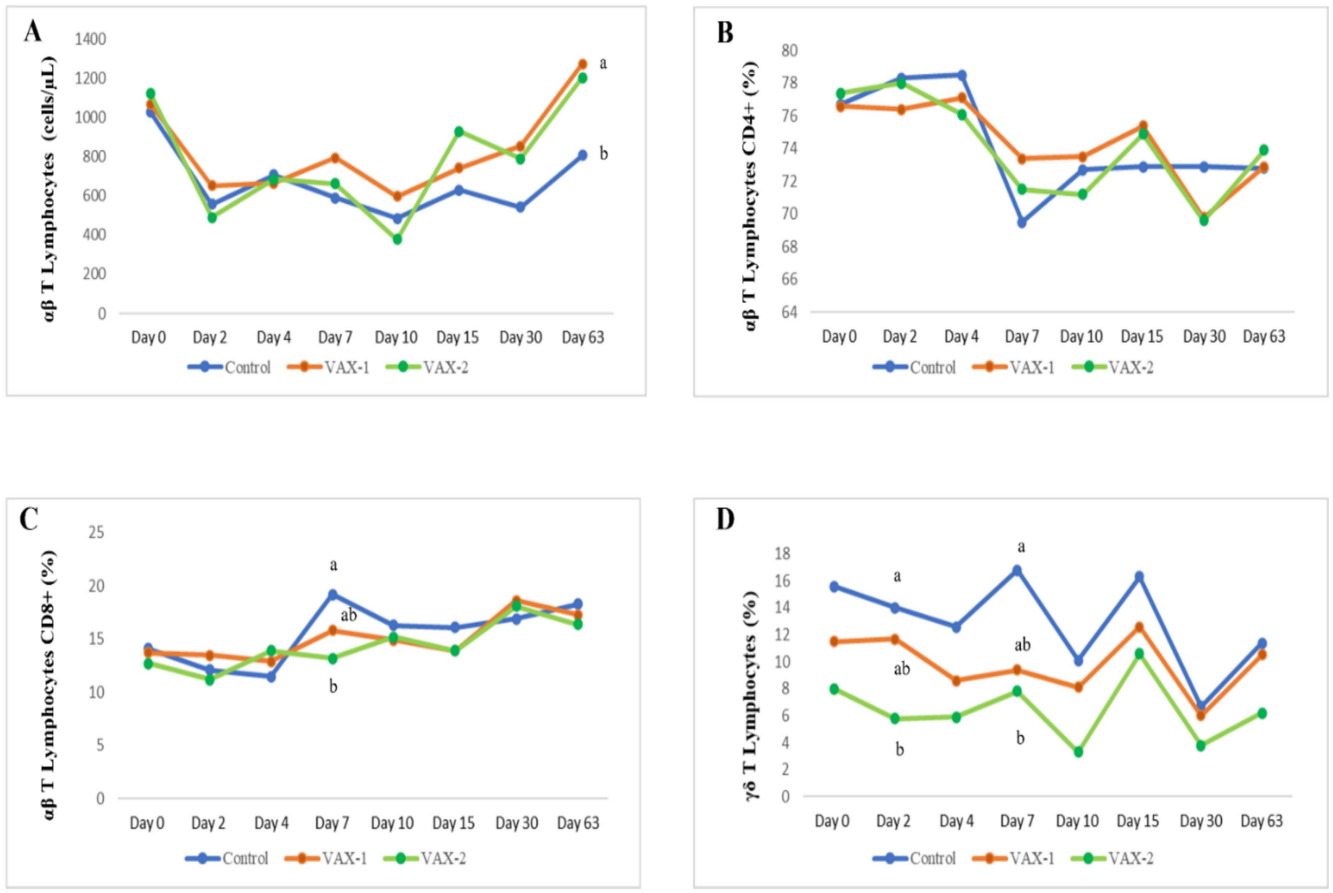
Time-related changes in the absolute count of T lymphocytes **(A)** and the percentage of αβ T CD4^+^ **(B)**, αβ T CD8^+^ **(C)** and γδ T cells **(D)** in vaccinated and unvaccinated buffalo calves after experimental infection with BAHV-1. Significant differences among groups at a single time point (a,b); *p* < 0.05.

Flow cytometric analysis was performed for this study to evaluate changes in B and T lymphocytes subsets in response to BuAHV-1 infection in water buffaloes vaccinated with two different marker vaccines. Supplementary Table S1 shows the differences in T and B lymphocytes subset between unvaccinated (CNT) and vaccinated (VAX-1 and VAX-2) groups during vaccination. In Table 6, the measurements of each lymphocyte subset during the whole period after experimental infection are shown. In addition, in Figure 2, values for each subset and at each time point were compared through groups. The results of the hematologic profile during infection with BuAHV-1 of the same groups of water buffaloes were reported in a previous paper (15). Flow cytometric analysis revealed that although the percentage of αβ T lymphocytes decreased significantly at an early stage only in control animals, the total αβ T lymphocyte count decreased also in both groups of vaccinated animals (Table 6). At PCD 63, lower values were observed in the CNT compared to vaccinated groups (Figure 2A). The percentage of CD4^+^ αβ T subsets showed a significant decrease from PCD 7 to PCD 63 for all groups, showing lower values than PCD 0. The differences were significant for all points only for the CNT group (Table 6). No significant differences among groups were observed at each time point (Figure 2B). The percentage of αβ T CD8^+^ showed a significant increase at PCD 30 for VAX 2 group, and at PCD 63 for all groups.” Moreover, differences between CNT and VAX-2 were observed at PCD 7 (Figure 2C). The αβ CD4/αβ CD8 ratio showed a significant decrease between PCDs 0 and 63 (Table 6). Regarding γδ T lymphocytes, a significant decrease in the total count was observed only in the CNT group at PCDs 4, 10, and 30. As for the percentage of γδ T lymphocytes, significantly lower values were noted at PCD 10 for the CNT and VAX 2 groups, at PCD 30 for the CNT and VAX 1 groups, and at PCD 63 for the CNT group (Table 6). Compared to the CNT group, both vaccinated groups evidenced lower values at each time point (Figure 2D). About B lymphocytes, no significant differences were noted in their percentage or total count across the various PCDs. However, a significant decrease in CD21^+^ B lymphocytes was observed in the CNT group at PCD 7, 10, 15, and 30 compared to PCD 0. In the VAX 1 group, significant differences were detected at PCD 15 and 30 (Table 6).

**Table 6:** Comparison of percentages and absolute counts (mean ± SEM) of lymphocyte subsets in CNT, VAX-1, and VAX-2 groups. Post-challenging values (at PCDs 2, 4, 7, 10, 15, 30 and 63) were compared to the pre-challenging values (at PCD 0) for each lymphocyte subpopulation.

	Post-challenge day (PCD)								
	Group	0^*^	2	4	7	10	15	30	63
		Mean ± SEM	Mean ± SEM	Mean ± SEM	Mean ± SEM	Mean ± SEM	Mean ± SEM	Mean ± SEM	Mean ± SEM
αβ T Lymphocytes (%)	CNT	**22.8 ± 2.5** ^**A**^	**15.8 ± 2.5** ^**B**^	18.3 ± 2.5	**13.3 ± 2.5** ^**B**^	**12.4 ± 2.5** ^**B**^	**16.0 ± 2.5** ^**b**-B^	**11.3 ± 2.5** ^**B**^	**13.6 ± 2.5** ^**B**^
	VAX-1	19.2 ± 2.7	13.4 ± 2.7	12.2 ± 2.7	13.8 ± 2.7	11.5 ± 2.7	16.3 ± 2.7b	11.8 ± 2.7	20.5 ± 2.7
	VAX-2	22.5 ± 2.7	10.8 ± 2.7	14.7 ± 2.7	11.9 ± 2.7	7.4 ± 2.7	**24.9 ± 2.7** ^**a**^	11.5 ± 2.7	17.7 ± 2.7
αβ T Lymphocytes (cells/uL)	CNT	**1,028 ± 124** ^**A**^	**558 ± 124** ^**B**^	707 ± 124^B^	**591 ± 124** ^**B**^	**485 ± 124** ^**B**^	**629 ± 124** ^**B**^	**542 ± 124** ^**B**^	**806 ± 124** ^**b**^
	VAX-1	**1,068 ± 139** ^**A**^	**652 ± 139** ^**B**^	**664 ± 139** ^**B**^	795 ± 139	**597 ± 139** ^**B**^	741 ± 139	853 ± 139	**1,273 ± 139** ^**a**^
	VAX-2	**1,123 ± 139** ^**A**^	**490 ± 139** ^**B**^	**685 ± 139** ^**B**^	661 ± 139^B^	**379 ± 139** ^**B**^	930 ± 139	790 ± 139	**1,203 ± 139** ^**a**^
αβ T CD4^+^ (%)	CNT	**76.7 ± 1.9** ^**A**^	78.3 ± 1.9	78.5 ± 1.9	**69.5 ± 1.9** ^**B**^	72.7 ± 1.9^B^	72.9 ± 1.9^B^	72.9 ± 1.9^B^	72.8 ± 1.9^B^
	VAX-1	**76.6 ± 2.2** ^**A**^	76.4 ± 2.2	77.1 ± 2.2	73.4 ± 2.2	73.5 ± 2.2	75.4 ± 2.2	**69.8 ± 2.2** ^**B**^	72.9 ± 2.2
	VAX-2	**77.4 ± 2.2** ^**A**^	78.0 ± 2.2	76.1 ± 2.2	**71.5 ± 2.2** ^**B**^	**71.2 ± 2.2** ^**B**^	74.9 ± 2.2	**69.6 ± 2.2** ^**B**^	73.9 ± 2.2
αβ T CD8^+^ (%)	CNT	**14.1 ± 1.8** ^**B**^	12.1 ± 1.8	11.5 ± 1.8	**19.2 ± 1.8** ^a-**A**^	16.3 ± 1.8	16.1 ± 1.8	16.9 ± 1.8	**18.3 ± 1.8** ^**A**^
	VAX-1	**13.7 ± 2.0** ^**B**^	13.5 ± 2.0	12.9 ± 2.0	15.8 ± 2.0^ab^	14.9 ± 2.0	13.9 ± 2.0	18.6 ± 2.0	**17.3 ± 2.0** ^**A**^
	VAX-2	**12.7 ± 2.0** ^**B**^	11.2 ± 2.0	13.9 ± 2.0	13.2 ± 2.0^b^	15.2 ± 2.0	13.9 ± 2.0	**18.1 ± 2.0** ^**A**^	16.4 ± 2.0^A^
αβ CD4/ αβ CD8	CNT	**5.53 ± 1.00** ^**A**^	6.62 ± 1.00	7.01 ± 1.00	3.70 ± 1.00	4.64 ± 1.00	4.82 ± 1.00	4.35 ± 1.00	**4.07 ± 1.00** ^**B**^
	VAX-1	6.06 ± 1.12^A^	6.37 ± 1.12	8.08 ± 1.12	5.90 ± 1.12	6.28 ± 1.12	5.93 ± 1.12	4.31 ± 1.12^B^	4.48 ± 1.12^B^
	VAX-2	6.23 ± 1.12^A^	7.32 ± 1.12	5.66 ± 1.12	5.52 ± 1.12	4.92 ± 1.12	5.53 ± 1.12	4.46 ± 1.12^B^	4.67 ± 1.12^B^
γδ T Lymphocytes (%)	CNT	**15.6 ± 2.8** ^**A**^	14.0 ± 2.8^a^	12.6 ± 2.8	16.8 ± 2.8^a^	**10.1 ± 2.8** ^**B**^	16.3 ± 2.8	**6.7 ± 2.8** ^**B**^	11.4 ± 2.8^B^
	VAX-1	11.5 ± 3.1^A^	11.7 ± 3.1^ab^	8.6 ± 3.1	9.4 ± 3.1^ab^	8.1 ± 3.1	12.6 ± 3.1	6.0 ± 3.1^B^	10.5 ± 3.1
	VAX-2	8.0 ± 3.1^A^	5.8 ± 3.1^b^	5.9 ± 3.1	7.8 ± 3.1^b^	3.3 ± 3.1^B^	10.6 ± 3.1	3.8 ± 3.1	6.2 ± 3.1
γδ T Lymphocytes (cells/μL)	CNT	**863 ± 176** ^**A**^	651 ± 176	**640 ± 176** ^**B**^	841 ± 176	**472 ± 176** ^**B**^	750 ± 176	**356 ± 176** ^**B**^	717 ± 176
	VAX-1	634 ± 197	543 ± 197	646 ± 197	532 ± 197	406 ± 197	564 ± 197	432 ± 197	659 ± 197
	VAX-2	408 ± 197	268 ± 197	273 ± 197	377 ± 197	165 ± 197	400 ± 197	261 ± 197	426 ± 197
γδ T WC1^+^ (%)	CNT	**92.4 ± 3.4** ^**A**^	92.8 ± 3.4	89.6 ± 3.4	**81.2 ± 3.4** ^**B**^	87.8 ± 3.4	91.8 ± 3.4	**80.4 ± 3.4** ^**B**^	87.4 ± 3.4
	VAX-1	**90.0 ± 3.8** ^**A**^	91.5 ± 3.8	85.3 ± 3.8	83.9 ± 3.8	85.6 ± 3.8	89.4 ± 3.8	**74.8 ± 3.8** ^**B**^	88.2 ± 3.8
	VAX-2	89.9 ± 3.8^A^	92.2 ± 3.8	80.6 ± 3.8^B^	82.7 ± 3.8	83.1 ± 3.8	90.3 ± 3.8	81.1 ± 3.8^B^	88.4 ± 3.8
B Lymphocytes (cells/μL)	CNT	0.82 ± 0.17	0.67 ± 0.17	0.72 ± 0.17	0.75 ± 0.17	0.67 ± 0.17	0.62 ± 0.17	0.80 ± 0.17	0.99 ± 0.17
	VAX-1	0.79 ± 0.19	0.67 ± 0.19	0.62 ± 0.19	0.60 ± 0.19	0.63 ± 0.19	0.58 ± 0.19	0.84 ± 0.19	0.81 ± 0.19
	VAX-2	0.75 ± 0.19^A^	0.54 ± 0.19^B^	0.58 ± 0.19	0.68 ± 0.19	0.56 ± 0.19	0.52 ± 0.19	0.80 ± 0.19	0.68 ± 0.19
B Lymphocytes (%)	CNT	33.4 ± 5.0	32.3 ± 5.0	30.8 ± 5.0	33.1 ± 5.0	28.5 ± 5.0	37.7 ± 5.0	30.6 ± 5.0	36.5 ± 5.0
	VAX-1	33.6 ± 5.6	34.0 ± 5.6	31.7 ± 5.6	30.9 ± 5.6	31.2 ± 5.6	33.1 ± 5.6	32.9 ± 5.6	30.0 ± 5.6
	VAX-2	32.5 ± 5.6	31.4 ± 5.6	30.8 ± 5.6	32.2 ± 5.6	29.4 ± 5.6	31.1 ± 5.6	32.1 ± 5.6	27.8 ± 5.6
CD21^+^ B (%)	CNT	**93.1 ± 2.5** ^**A**^	89.3 ± 2.5	89.9 ± 2.5	**86.6 ± 2.5** ^**B**^	**86.7 ± 2.5** ^**B**^	**87.4 ± 2.5** ^**B**^	**87.5 ± 2.5** ^**B**^	91.3 ± 2.5
	VAX-1	92.0 ± 2.8^A^	90.0 ± 2.8	90.8 ± 2.8	90.2 ± 2.8	90.8 ± 2.8	87.6 ± 2.8^B^	86.8 ± 2.8^B^	91.9 ± 2.8
	VAX-2	91.2 ± 2.8	88.0 ± 2.8	90.4 ± 2.8	86.0 ± 2.8	90.3 ± 2.8	89.4 ± 2.8	87.4 ± 2.8	86.6 ± 2.8

^*^PCD 0 corresponds to PVD 55; Significant differences between groups at each single timepoint (^a,b^); significant differences between PCD 0 and a single timepoint (^A,B^); *p* < 0.05. Bold significant differences *p* < 0.01–0.001.

## 4 Discussion

The European Union (EU) Animal Health Regulation (16) and the EU Delegated Regulation (18) have been adopted to ensure that each Member State is officially (fully or partially) free from IBR. In accordance with the Delegated Regulation (EU) 2018/1882, IBR/IPV is listed in categories C, D and E, and *Bubalus ssp*. is a susceptible species. Furthermore, the strategy of differentiating vaccinated from infected animals (also called DIVA) has been implemented (18). Based on the serological cross-reactivity of BoAHV-1 and BuAHV-1, we hypothesized that the use of an IBR marker vaccination protocol to immunize cattle against BoAHV-1 would be able to induce protection in water buffaloes following infection with BuAHV-1 (19). This hypothesis was tested by evaluating two commercial IBR marker vaccines. The products used for cattle have shown efficacy in reducing the incidence of gE seroconversion in dairy cattle and, consequently, the prevalence in the herd. In addition, they have demonstrated protection against BoAHV-1 infection, including a strong immune response. Furthermore, live or inactivated IBR marker vaccines have demonstrated efficacy in cattle (13, 20).

The results obtained in this study showed that the vaccines tested caused no clinical signs or adverse reactions. The data from this study are consistent with previously reported studies in cattle and buffalo (19, 22–24), suggesting no risk of adverse reactions after vaccine administration using the proposed protocol.

Rectal temperature remained at normal physiological levels after infection in both groups of vaccinated animals. Furthermore, none of the immunized animals showed any clinical signs of disease throughout the experimental period. In contrast, four control animals showed nasal mucous discharge; one showed a nasal mucous membrane lesion associated with mucous exudate and increased rectal temperatures (39.1°C). These clinical findings differ from Scicluna et al. (9), who observed no clinical signs. Two other studies described similar results. In particular, Petrini et al. (13) reported rhinorrhea, pseudomembranes, respiratory distress and trapping in 3/5 animals, and Montagnaro et al. (21) described rhinorrhea in 5/5 animals.

Nasal swabs showed positivity by gB real-time PCR in all groups after PCD 2, but for a shorter time in vaccinated animals (up to PCD 7) than in control animals (up to PCD 15). However, these results differ from our previous study (13), in which vaccinated animals did not shed the virus used for challenge infection, unlike the control group that shed the virus up to PCD 7 (13). These results are probably due to the fact that the animals infected in the present study were younger (6 months) than those infected by Petrini et al. (13) (17 months). The age of the water buffaloes used in this study may affect a host’s immune response.

The gB-ELISA and the NA (BuAHV-1-BoAHV-1) were positive for the first time on PVD 14. The same antibodies were observed in the vaccinated group up to the end of the experiment compared to the control group. Studies using modified live vaccines (MLV) or inactivated IBR marker vaccines (gE-) reported similar results (21). In addition, the serological results were in line with previous studies in cattle immunized with IBR marker vaccines (gE-) (22–25). During the vaccination period, immunized water buffaloes showed negative gE-ELISA results. This outcome indicates that BoAHV-1 and BuAHV-1 were not circulating during the experimental period.

In the present study, we detected gE-ELISA positivity in water buffaloes vaccinated at PCD 63, whereas gE-ELISA seroconversion was observed in the control group at PCD 15. These results obtained in water buffalo are similar to those evidenced in cattle (26, 27). In contrast, no seroconversion in water buffalo following challenge infection was reported by Montagnaro et al. (21). The results obtained by Montagnaro et al. may be due to the shorter duration of post-challenge evaluation (15 days), in which the animals may not have had time to become seropositive.

This study showed that the vaccinated animals shed the virus up to PCD 7, indicating that the vaccination protocol tested did not protect the animals against wt-BuAHV-1 infection. However, the presence of antibody titers detected by VN after vaccination and their subsequent rise after challenge are indicators of possible protection. Reducing challenge virus replication, which should result from the sum of humoral and cellular immune responses, would be the true evidence of protective immunity.

The differences observed in the immune responses between cattle and buffalo can be attributed to animal genetics, the geographical location of the farm, weather, nutrition, health status of the herd, circulating vaccine strains and viral concentration involved. Furthermore, although the experimental period after the challenge was not very long, it is generally known that the immune response evoked following herpetic infections lasts for months/years. In addition, the serological data obtained in this study are not comparable with those obtained from a possible vaccine registered for the buffalo species; in fact, to date, no vaccine is available on the world market.

Moreover, this study focuses on the time-related circulatory kinetics of B and T lymphocyte subsets in water buffalo calves induced by vaccination and following BuAHV-1 infection. Lymphocytes are two broad classes of adaptive immune responses: the cell-mediated immune response (T lymphocytes) and the antibody response (B lymphocytes). These populations are the main players in the adaptive immune response to invading viruses and bacteria (28). It is known that mammalian T cells are characterized by a T cell receptor (TCR), responsible for recognizing antigens presented by MHC molecules. Despite the cellular immune response to Alphaherpesviruses infection in water buffalo has been poorly investigated, the quantification of lymphocytes and monocyte subsets in peripheral blood during BoAHV-1 and BuAHV-1 infections has been recently performed by flow cytometric analysis (13, 15). Results from this study showed that after the challenge infection with BuAHV-1, all groups experienced a decrease in relative and absolute count of αβ T lymphocytes, starting from day 2 until day 10, mainly due to the decrease of αβT CD4^+^ subset. However, a sharp decline was observed in unvaccinated water buffaloes, which continued until PCD 63. At this time, lymphopenia was more evident in the control group than in both vaccinated groups. This result is consistent with the reported lymphopenia associated with BoAHV-1 infection in calves (29) and in agreement with our previous study (13), which showed a recovery of T lymphocytes PCD 63. Eskra et al. (30) evidenced that BoAHV-1 selectively infects CD4^+^ T cells and, to a lesser extent, CD8^+^ T cells, inducing apoptosis and impairing the host immune responses (31). Likewise, an increase of CD8^+^ T cells was observed in the CNT group at PCD 7 and for all groups at PCD 63. The γδ T cells represent 60% of total circulating T lymphocytes in adult ruminants, including cattle (32). These subsets of T lymphocytes, activated through TCRs in response to invading pathogens, release cytokines, pro-inflammatory and anti-inflammatory mediators in the framework of a regulatory/suppressive activity reviewed by Righi et al. (33). As regards the percentage and total count of γδ T cells, for the whole period of infection, higher values were observed in the CNT group compared to both vaccinated groups, with significant differences between groups only at PCDs 2 and 7. Previous studies showed that the quantification of cell subsets in peripheral blood during BoAHV-1 infection was different depending on the vaccination protocol used (34–37).

Therefore, the results of the present study suggest paying more attention to the vaccine administration protocol. Finally, regarding the B lymphocyte population, only the CD21^+^ subset showed a decreasing trend during the time course of infection in the CNT group. Overall, the flow cytometric results indicated that BuAHV-1 activates the adaptive immune responses, and in addition to the fact that vaccinated and unvaccinated animals showed the same trend throughout the examination period, significant differences were observed on CD4^+^, CD8^+^ and γδ T cells. Perhaps the use of a vaccine dosage for cattle and not for water buffaloes may have influenced the results. Therefore, further field studies will be needed to evaluate new vaccination protocols for water buffaloes to successfully include a vaccine in the eradication of IBR.

## 5 Conclusion

In conclusion, the results of this study allow us to continue the investigation regarding the immune system of the water buffalo species in even greater detail. The results demonstrated that vaccination in the water buffalo species with IBR marker vaccines (gE-/tk-; gE-) authorized in cattle is innocuous and efficacious. The studies conducted in this research are preliminary to a control plan for IBR developed in Italy. In addition, further field studies are needed to evaluate safety and efficacy of these IBR marker vaccines in the water buffalo species on a large scale before their application.

## Data Availability

The original contributions presented in the study are included in the article/Supplementary material, further inquiries can be directed to the corresponding author.

## Supplementary material

SUPPLEMENTARY TABLE S1 Comparison of T and B lymphocytes subset between group of calves unvaccinated (CNT) and vaccinated with two different IBR marker vaccines (VAX-1 and VAX-2) during the time course of vaccination.
