# Supplementary material for: Evaluation of two commercial IBR marker vaccines against Bubaline alphaherpesvirus 1 in water buffalo (Bubalus bubalis)
Source: Front Vet Sci. 2025 May 7;12:1574794. doi: 10.3389/fvets.2025.1574794 (PMC12094042; doi:10.3389/fvets.2025.1574794)
Supplement: SUPPLEMENTARY TABLE S1 — Comparison of T and B lymphocytes subset between group of calves unvaccinated (CNT) and vaccinated with two different IBR marker vaccines (VAX-1 and VAX-2) during the time course of vaccination. [file Table_1.pdf]

Table S1: Comparison of T and B lymphocytes subset between group of calves unvaccinated (CNT) and vaccinated with two different marker vaccines (VAX-1 and VAX-2) during the time course of vaccination (PVD).

	**Group**	**PVD**			
		**0**	**14**	**20**	**34**
		**(Mean ± SEM)**	**(Mean ± SEM)**	**(Mean ± SEM)**	**(Mean ± SEM)**
αβ T lymphocytes (%)	CNT	14.51±3.77	22.90±3.77	15.81±3.77	20.78±3.77
	VAX-1	13.48±4.22	24.50±4.22	16.08±4.22	20.45±4.22
	VAX-2	15.67±4.22	24.26±4.22	15.32±4.22	22.42±4.22
αβ T lymphocytes (cells/uL)	CNT	893±183	945±183	776±183	1223±183
	VAX-1	897±205	1058±205	990±205	1401±205
	VAX-2	973±205	1181±205	810±205	1419±205
αβ $CD4^+$ T (%)	CNT	75.12±2.03	75.71±2.03	76.74±2.03	74.34±2.03
	VAX-1	74.83±2.27	74.70±2.27	75.70±2.27	77.98±2.27
	VAX-2	75.60±2.27	70.94±2.27	74.02±2.27	77.59±2.27
αβ $CD8^+$ T (%)	CNT	15.62±1.61	16.25±1.61	14.82±1.61	16.51^A±1.61
	VAX-1	16.61±1.80	16.5±1.80	15.13±1.80	12.37^B±1.80
	VAX-2	14.31±1.80	13.98±1.80	15.24±1.80	12.06^B±1.80
CD4/CD8	CNT	4.88±1.21	4.74±1.21	5.38±1.21	4.73^b±1.21
	VAX-1	4.54±1.36	4.9±1.36	5.36±1.36	8.36^a±1.36
	VAX-2	5.38±1.36	5.25±1.36	5.31±1.36	6.56^a±1.36
γδ T lymphocytes (%)	CNT	9.97±3.16	16.21±3.16	12.7±3.16	15.62±3.16
	VAX-1	8.19±3.54	19.08±3.54	12.97±3.54	11.97±3.54
	VAX-2	7.77±3.54	12.76±3.54	8.04±3.54	9.35±3.54
γδ T lymphocytes (cells/uL)	CNT	749±206	874±206	692±206	1062±206
	VAX-1	563±230	763±230	812±230	817±230
	VAX-2	496±230	611±230	430±230	613±230
γδ T $WC1^+$ (%)	CNT	89.57±1.32	92.65±1.32	94.00±1.32	94.01^a±1.32
	VAX-1	85.25±1.48	90.51±1.48	90.75±1.48	88.62^b±1.48
	VAX-2	90.98±1.48	91.44±1.48	91.54±1.48	90.85^a±1.48
B Lymphocytes $CD21^+$ (%)	CNT	91.94±1.59	92.82±1.59	92.87±1.59	93.14±1.59
	VAX-1	92.61±1.78	92.49±1.78	89.99±1.78	91.14±1.78
	VAX-2	89.56±1.78	90.95±1.78	90.95±1.78	91.54±1.78
B Lymphocytes $CD21^-$ (%)	CNT	7.78±1.56	6.79±1.56	6.79±1.56	6.53±1.56
	VAX-1	7.13±1.75	7.18±1.75	9.61±1.75	8.31±1.75
	VAX-2	10.06±1.75	8.78±1.75	8.80±1.75	8.09±1.75

a, b= significant difference between groups with $p <$ 0.01-0.001 A, B= trend of significance with P< 0.10.
